# Characterization of the key membrane protein cytochrome Cyc2 from *Ghiorsea bivora*

**DOI:** 10.1002/2211-5463.70350

**Published:** 2026-10-23

**Authors:** Josiane Azizi, Alain Dolla, Frauke Baymann, Celine Rommevaux, Latifa Elantak, Helene Gaussier, Matthieu Nouailler

**Affiliations:** ^1^ Aix Marseille University, Université de Toulon, CNRS, IRD, MIO Marseille France; ^2^ Aix Marseille University, CNRS, LISM Marseille France; ^3^ Aix Marseille University, CNRS, BIP Marseille France

**Keywords:** Cyc2, cytochrome c, ferroxidase activity, *Ghiorsea bivora*, iron oxidation, membrane enzyme, redox potential, Zetaproteobacteria

## Abstract

This study reports the heterologous production, purification, and characterization of the cytochrome Cyc2_TAG1_ cluster 1 (Cyc2_TAG1_1_) from *Ghiorsea bivora* TAG1 in the outer membrane of *Escherichia coli* BL21. Using a specific gene construct we optimized solubilization and purification protocols to obtain high‐purity enzyme. This enabled biophysical characterization by EPR, measurement of its midpoint redox potential, and the development of an iron‐oxidizing assay with 2,6‐dichlorophenolindophenol (DCPIP) as an artificial final electron acceptor. The study provides the first quantitative kinetic characterization of a Cyc2 homolog *in vitro*. The absence of comparable apparent K_m_ or V_max_ values with a natural or artificial electron acceptor in the literature underscores the novelty of our findings. Cyc2_TAG1_1_ exhibited an apparent K_m_ of 627 ± 226 μm for iron sulfate, consistent with iron‐rich hydrothermal vent environments where *G. bivora* thrives, and an apparent V_max_ of 19 ± 5 μm·s^−1^. Despite using an artificial electron acceptor that does not accurately represent the natural partner or the real energetic system, the enzyme exhibits apparent kinetic parameters that retain biological significance, with a catalytic efficiency within the range expected for functional electron transfer enzymes. Cyc2_TAG1_1_ exhibited a redox potential of −130 ± 20 mV *vs* SHE which is the lowest reported for Cyc2 homologs. The EPR spectrum is consistent with a low‐spin ferric heme with two axial ligands within the protein barrel. These findings provide the first biochemical characterization of Cyc2_TAG1_1_ and support its proposed role in iron oxidation.

## Graphical abstract

This study reports the first detailed biochemical analysis of Cyc2TAG1_1, a cytochrome protein involved in microbial iron oxidation. We produced and purified this enzyme in *E. coli*, then measured its iron‐oxidizing efficiency, and characterized its electrical and structural properties. These results clarify how iron‐oxidizing bacteria harvest energy and pave the way for studying Cyc2TAG1_1 under more physiologically relevant conditions.

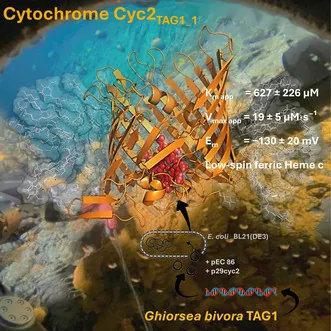

**Abbreviations**
APBS: Adaptive Poisson–Boltzmann Solver
BIF: Banded Iron Formation
CMC: Critical Micellar Concentration
DCPIP: 2,6‐Dichlorophenolindophenol
DDM: N‐Dodecyl‐β‐D‐maltoside
EDTA: Ethylenediaminetetraacetic Acid
EPR: Electron Paramagnetic Resonance
FeOB: Ferrous Iron‐Oxidizing Bacteria
HEPES: 4‐(2‐Hydroxyethyl)‐1‐piperazineethanesulfonic Acid
IPTG: Isopropyl‐β‐D‐1‐thiogalactopyranoside
NLS: N‐Lauroylsarcosine
NTA: Nitrilotriacetic Acid
OM: Outer Membrane
SDS/PAGE: Sodium Dodecyl Sulfate–Polyacrylamide Gel Electrophoresis
SHE: Standard Hydrogen Electrode
TAG: Trans‐Atlantic Geotraverse
TMBZ: 3,3′,5,5′‐Tetramethylbenzidine
ZetaOTU: Zetaproteobacteria Operational Taxonomic Unit

In marine ecosystems, iron plays a key role in regulating primary productivity and carbon cycling. It is needed by phytoplankton for respiration, nitrogen fixation and is an essential micronutrient for photosynthesis [1]. Its main sources are hydrothermal vents together with other anthropogenic or natural sources such as mixing with sediments, aerosol dust solubilization, fluxes through rivers, and cryospheric meltwaters [2]. In seawater, iron exists primarily in two oxidation states: the more soluble ferrous iron Fe(II) which predominates in anoxic environments and the less soluble ferric iron Fe(III). Fe(II) is highly reactive to changes in pH, oxygen concentrations, complexing ligands, and photochemical conditions. In oxic surface waters, Fe(II) oxidizes abiotically to Fe(III) resulting in the formation of Fe(III) oxyhydroxide precipitates commonly referred to as rust. However, iron oxidation is not only a chemical process; biotic iron oxidation carried out by anoxygenic phototrophs and microaerophilic iron‐oxidizing bacteria represents an ancient and active metabolism that has persisted for over a billion years, particularly when oceans were rich in Fe(II) and low in O_2_ [3]. Both iron oxidations are predicted to have a role in the formation of the Banded Iron Formation (BIF) during the Precambrian stage [4, 5].

Ferrous iron‐oxidizing bacteria (FeOB) constitute a broad group, belonging mainly to the Proteobacteria phylum, that shares the ability to use Fe(II) as an electron donor. Out of all the species found in the ocean, most of them fall into one class, the Zetaproteobacteria [6]. Zetaproteobacteria are chemolithotrophic bacteria that derive energy from iron oxidation at circumneutral pH and low oxygen concentrations (micromolar to submicromolar range) where there is less competition with abiotic iron oxidation (Reaction 1) [7].

(1) $$\text{4Fe}\left(\mathrm{II}\right)+10H_{2}O+O_{2}\to \text{4Fe}{\left(\mathrm{OH}\right)}_{3}+{\text{8H}}^{+}$$

Zetaproteobacteria frequently produce different types and forms of iron oxides that can be associated with other organic components such as exopolysaccharides and form a few centimeters thick iron‐rich microbial mats [8, 9, 10]. *Mariprofundus ferrooxydans* (strains PV‐1 and JV‐1), the first isolated strains of Zetaproteobacteria, were recovered from an Fe(II)‐rich hydrothermal system at Loihi (Kama'ehuakanaloa) Seamount in Hawaii [11].

Within Zetaproteobacteria, *Ghiorsea bivora* (TAG‐1 and SV‐108) is a recently reported genus and species, isolated from an iron‐rich microbial mat in the Trans‐Atlantic Geotraverse (TAG) hydrothermal area along the Mid‐Atlantic Ridge [12]. Contrary to other known zetaproteobacterial iron oxidizers, *G. bivora* can grow using either Fe(II) or H_2_ as an energy source. It is currently the sole cultured representative of Zetaproteobacteria operational taxonomic unit 9 (ZetaOTU 9), a widespread and abundant lineage found in iron‐rich deep‐sea habitats, including hydrothermal vents and the marine subsurface [13, 14].

In FeOB, the electron transport chain from Fe(II) was initially suggested and studied in the acidophilic iron‐oxidizing bacteria *Acidithiobacillus ferrooxidans* [15, 16]. Since then, many research has been carried out on it [17, 18, 19]. Electrons from Fe(II) are transferred ‘downhill’ to oxygen (O_2_) via an ‘electron wire’ spanning both the outer and inner membranes, with the outer membrane c‐type cytochrome Cyc2 constituting the first proposed enzyme for Fe(II) oxidation. Castelle *et al*. provided the first *in vitro* validation of the iron‐oxidizing activity of the outer membrane cytochrome Cyc2 from *A. ferrooxidans* and measured its redox potential, reported at +560 mV at pH 4.8, the highest measured for a cytochrome c [19].

In order to identify the potential Fe(II) oxidase in Zetaproteobacteria, Barco *et al*. [20] performed a proteomic study on *M. ferrooxydans* PV‐1 leading to the identification of the iron oxidase Cyc2 and other proteins, through a proteome comparison with the closely related *M. ferrooxydans* genome. The identified Cyc2 protein in PV‐1 is closely related to the experimentally characterized Cyc2 Fe(II)‐oxidase from *A. ferrooxidans* [19], sharing 22% amino acid sequence identity [20]. The alignment of Cyc2‐like proteins from neutrophilic FeOB with Cyc2 from acidophilic FeOB reveals homology at the N terminus, highlighted by several conserved amino acids, including a conserved heme‐binding motif. Although the protein core lacks significant sequence homology, it is consistently predicted to form a beta‐barrel structure [20, 21, 22]. Metagenomic and metatranscriptomic studies on iron mat samples have identified Cyc2 homologs in other Zetaproteobacteria and diverse neutrophilic FeOB, further supporting the proposed pathway [23, 24].

Phylogenetic analysis revealed that Cyc2 sequences (from terrestrial to marine and circumneutral to acidic environments) cluster into three major groups, based on their conserved regions: Cluster 1, 2, and 3. Members of the Zetaproteobacteria possess Cyc2 proteins from both Clusters 1 and 3, although the Cluster 3 *cyc2* gene is present in fewer ZOTUs. This distribution suggests that both Cyc2 clusters play functional roles in Zetaproteobacteria. However, the specific functional differences between Cluster 1 and Cluster 3 Cyc2 proteins remain unclear [24]. Other components of the respiratory chain such as terminal cytochrome oxidase as well as other key enzymes were also identified [24].

In 2021, Keffer *et al*. validated the iron‐oxidizing activity of Cyc2_PV‐1_ cluster 1. Their study reported the first heterologous production and purification of Cyc2_PV‐1_ from *M. ferrooxydans* in *E. coli* enabling direct confirmation of its iron‐oxidizing activity *in vitro* and determination of its redox potential. At pH 8, Cyc2_PV‐1_ exhibits a redox potential of +208 mV ± 20 mV *vs* Standard Hydrogen electrode (SHE), placing it in the bioenergetic redox landscape of *M. ferrooxydans* [25]. As for *G. bivora* TAG1, two copies of Cyc2 belonging respectively to cluster 1 and 3 were also identified, but neither their iron oxidation activity nor any differential efficiency between them has been demonstrated to date [26]. Recently, a novel gene operon has been discovered in *G. bivora*, the *grc* operon, that seems to encode proteins with detectable similarities with cytochrome Cyc2, specially at the heme conserved domain, and may play a role in iron oxidation [26].

Owing to its ability to perform chemolithotrophy using hydrogen in addition to iron, its status as the sole representative of the widespread ZetaOTU9, and its broad ecological distribution, *Ghiorsea bivora* represents a highly relevant model organism for studying marine iron oxidation. Characterizing *G. bivora*'s Cyc2_TAG1_ cluster 1 will enable a better understanding of its activity as an iron oxidase in the respiratory chain of FeOB from iron‐rich marine ecosystems. The goal of this work is to produce this challenging membrane protein, which requires a bacterial model adapted for correct membrane insertion and a specialized enzyme system for heme *c* maturation. We also seek to extend the biochemical characterization of *G. bivora*'s Cyc2_TAG1_1_ by measuring its kinetic parameters, EPR spectrum, and its redox potential.

## Material and methods

### Cloning and heterologous expression of *cyc2*_*TAG1_1*_ gene

A fusion *cyc2*_*TAG1*_ gene (WP_038247608.1) from Cyc2_TAG1_1_ cluster 1 of *G. bivora* (NCBI accession number: JQLW00000000) was constructed. SignalP analysis identified the native signal sequence and predicted its cleavage site at amino acid 22. The native cyc2 signal sequence was replaced at the 5′ end of the gene with that of *ompA*, the outer membrane protein A signal peptide from *Escherichia coli*. The sequences encoding a Tobacco Etch Virus (TEV) protease cleavage site, a linker region and a 7‐His tag were added at the 3′ end of the gene (Fig. S1). This construction was synthetized (IDTDNA Inc.) directly into pET‐29b(+) to give plasmid p29cyc2. *Escherichia coli* BL21(DE3) was then cotransformed by heat shock with p29cyc2 and pEC86, a plasmid carrying the cytochrome c maturation (*ccmABCDEFGH*) genes under the control of a constitutive promoter [27], to ensure proper heme maturation and insertion. Transformants were selected on LB agar medium supplemented with chloramphenicol (30 μg·ml^−1^) and kanamycin (100 μg·ml^−1^).

For heterologous production of the cytochrome Cyc2_TAG1_1_, *E. coli* BL21(DE3) (p29cyc2) (pEC86) was grown aerobically at 37 °C with shaking at 180 rpm in M9 2× media (Table S3) supplemented with both antibiotics. When cultures reached an optical density at 600 nm (OD_600_) of 0.6, fusion gene expression was induced by adding 0.5 mm isopropyl‐b‐D‐1‐thiogalactopyranoside (IPTG), 2 mm MgCl_2_, and 100 μm ferric citrate. The cells were then cultured overnight at 16 °C with shaking at 80 rpm.

### Purification of heterologous produced Cyc2_*TAG1_1*_

Recombinant *E. coli* cells (1 L culture) were harvested by centrifugation (3470 *g*) for 20 min at 4 °C, and the pellets were stored at −20 °C. For cell lysis, pelleted cells were washed and resuspended in 9 mL buffer A (50 mm triethanolamine, 250 mm sucrose, 1 mm EDTA (pH 7.5)) supplemented with protease inhibitor cocktail Complete EDTA‐free (Roche Diagnostics France, Meylan, France), then passed twice through a French pressure cell at 8000 psi. Unbroken cells and debris were removed by centrifugation for 15 min at 3220 *g*. The resulting supernatant was centrifuged for 4 min at 137 100 *g* at 4 °C to obtain the enriched outer membrane (OM) fraction pellet [28].

The OM enriched pellet fraction was solubilized by rotative shaking in 2 mL of buffer B (50 mm HEPES (4‐(2‐hydroxyethyl)‐1‐piperazineethanesulfonic acid) pH 7, 2% NLS) for 1 h at room temperature and unsolubilized material was then pelleted by ultracentrifugation (137 100 g) for 1 h at 4 °C. About 500 μL of the solubilized membrane was loaded onto a 4 mL Ni‐NTA Agarose XL beads equilibrated with 50 mm HEPES pH 7–0.2% NLS (equilibration buffer) and incubated for 1 h at room temperature with slow shaking. Ni‐NTA Agarose XL beads were then centrifuged at 2415 g for 15 s and successively washed 2 times with 40 mL of equilibration buffer. One mL of 500 mm imidazole in equilibration buffer was added to the Ni‐NTA beads, transferred to small column and bound proteins were eluted by gravity.

For the detergent exchange, the eluted fraction was diluted to 0.05% of NLS with buffer C (50 mm HEPES pH7–0.2% DDM) and incubated overnight at 4 °C. The diluted fraction was then concentrated on a 100 kDa cutoff concentrator (Sartorius Vivaspin 20 100 000 MWCO PES) by centrifugation at 2093 *g* at 4 °C for 5 min each cycle, mixing in between in order to minimize detergent concentrations as described by Gobet *et al*. [29], until reaching 1 mL of final volume. The fraction was then dialyzed against 50 mm HEPES pH7 overnight at 4 °C to remove residing NLS and imidazole. Cyc2_TAG1_1_ concentration was determined using the molar extinction coefficient of 29.5 mm ^−1^·cm^−1^ at 550 nm in the sodium dithionite reduced state [30, 31]. The purified cytochrome Cyc2_*TAG1*_ was stored at 4 °C for subsequent analyses.

### SDS/PAGE and heme staining

For analysis of cytochrome Cyc2_TAG1_1_ production in *E. coli*, 1 mL of overnight culture was pelleted by centrifugation at 12 100 g for 5 min and cells were chemically lysed by resuspension in 20 μL of Bacterial Protein Extraction Reagent (Thermo Fisher Scientific, Illkirch, France) for 20 min at room temperature. The protein content was analyzed by using sodium dodecyl sulfate 12% polyacrylamide gel electrophoresis. Cyc2_TAG1_1_ quantitation in the gel was performed by imagej.

For heme staining, the gel was immersed for 20 min in 70 mL of 0.25 m sodium acetate buffer (pH 5) with constant shaking in the dark [32, 33]. Then, 3 parts of freshly prepared 3,3′,5,5,5‐tetramethylbenzidine (TMBZ) solution in methanol (0.8 mg·ml^−1^) were mixed with 7 parts of 0.25 m sodium acetate. The gels were immersed in this solution at room temperature, in the dark, with constant shaking. After 20 min, 0.5 mL of hydrogen peroxide (H_2_O_2_) was added; heme‐containing bands appeared as blue bands within 3 min.

### Iron‐oxidizing activity test and kinetic parameters measurement

The iron‐oxidizing activity of cytochrome Cyc2_TAG1_1_ was assessed under anaerobic conditions in an anaerobic chamber (Coy, under 5% H_2_, 95% N_2_ atmosphere) at 30 °C using a SHIMADZU UV‐2600i spectrophotometer (tacking part in the glove box) by monitoring the reduction of DCPIP, which is widely recognized as an artificial electron acceptor [26]. The reaction was initiated by the addition of 100 μm of ferrous sulfate heptahydrate (Fe(II)) as the electron donor in a reaction medium containing 0.03 μm Cyc2_TAG1_1_ in buffer C (50 mm HEPES pH 7.0, 0.2 mm DCPIP, and 0.01% DDM). The final reaction volume was 100 μL. DCPIP reduction was monitored by following the decrease of the absorbance at 522 nm [34] over a 60‐s period with intervals of 0.2 s for 1 min.

The initial reaction rates (V₀) of Cyc2_TAG1_1_ were determined from the linear portion of the absorbance‐versus‐time curves recorded during the DCPIP reduction assay. The rates were converted to m·s^−1^ using the molar extinction coefficient of DCPIP at 522 nm (ε = 6800 m ^−1^·cm^−1^) [35]. All controls (Fe(II) + DCPIP and Cyc2_TAG1_1_ + DCPIP) and activity assays (Fe(II) + DCPIP+ Cyc2_TAG1_1_) were performed in parallel and in triplicate, and mean values were used for kinetic calculations. For kinetic parameters measurement, different iron concentrations ranging from 50 μm to 300 μm were used in the presence of 0.2 mm DCPIP. At Fe(II) concentrations exceeding 300 μm, DCPIP becomes the limiting reagent in our reaction. The iron oxidation rates measured in the controls (Fe(II) + DCPIP) were then subtracted from the corresponding sample rates at each Fe(II) concentration to measure the Cyc2_TAG1_1_ enzymatic activity. Based on these values, the apparent kinetic parameters (K_m_ and V_max_) were estimated using the hyperbolic Michaelis–Menten fit with nonlinear regression that were performed with R software (version 4.3.3.) (Data S1).

### Redox potential of the cytochrome Cyc2_*TAG1_1*_

A chemical titration was performed using sodium dithionite as the reductant and potassium ferricyanide as the oxidant in a 1 × 1 cm quartz cuvette at room temperature. The cuvette was purged with argon and maintained under an argon atmosphere with continuous stirring to ensure homogeneous mixing as described previously [36]. A combined Ag/AgCl electrode (Mettler Toledo) was used as the reference and connected to a potentiometer for measurement of the potential at each titration point. The electrode was calibrated before sealing it into the cuvette by placing it into a saturated solution of quinhydrone in 1.0 m HEPES buffer pH 7.0 [37]. The titration cell was installed in the sample compartment of the Cary 4000 spectrometer and spectra were recorded from 700 nm to 390 nm. About 10 μm cytochrome Cyc2_TAG1_1_ protein solution in 50 mm HEPES buffer containing 0.02% DDM and 50 mm KCl (pH 7.0) was mixed with the following redox mediators at a final concentration of 20 μM: para‐benzoquinone (redox midpoint potential: +280 mV), 2,5‐dimethyl‐p‐benzoquinone (+180 mV), 1,2‐naphthoquinone (+145 mV), phenazine methosulfate (+80 mV), phenazine ethosulfate (+55 mV), cresyl blue (+47 mV), duroquinone (+5 mV), 2,5‐dihydroxy‐p‐benzoquinone (−60 mV), and 2‐Hydroxy‐1,4‐naphthoquinone (−145 mV).

The cytochrome Cyc2_TAG1_1_ was reduced by successive additions of dithionite by microliter injections of a 200 mm dithionite solution. After each injection, the sample was stirred and the system was allowed to equilibrate for several minutes until no further changes were observed in the optical spectrum, indicating complete equilibration of the sample. The titration continued until further addition of dithionite did not result in spectral changes, and subsequently, the sample was reoxidized in a stepwise manner by ferricyanide. The procedure was repeated twice.

For data analysis, the spectrum of the fully reduced protein obtained at −225 mV was used as reference to calculate the reduced‐oxidized difference spectra (Fig. S2). The redox titration was evaluated by monitoring changes in the Soret band. The absorbance difference between the peak at 418 nm and the isosbestic point at 430 nm was calculated for each spectrum and plotted against the solution potential. The data were then fitted to a one‐electron (*n* = 1) Nernst equation using Origin.

### EPR

EPR spectra were recorded at 15 K with a microwave power of 6.5 mW, a microwave frequency of 9.468 GHz and a modulation amplitude of 3.15 mT with a Bruker Elexsys E500X‐band EPR spectrometer at 9.48 GHz fitted with an Oxford Instruments cryostat (ESR900) for temperature control and a rectangular standard cavity (ER4102ST, Bruker France S.A.S., Wissembourg, France). EPR spectra were recorded on 15 μm of nondialyzed purified cytochrome Cyc2_TAG1_1_ and on the enrichment of outer membrane.

### Structure prediction

For AI‐based prediction of the cytochrome Cyc2_TAG1_1_, AlphaFold 3 tool by Google DeepMind was used for structural predictions [38]. Cyc2_TAG1_1_ sequence residues 23–389 (black and underlined residues in Fig. S1) served as input for structure prediction. SignalP analysis identified the native signal sequence responsible for outer membrane targeting and predicted a cleavage site after residue 22. Accordingly, residues 1–22 were removed prior to AlphaFold modeling, and structure prediction was performed using the mature protein sequence starting from residue 23. The heme c cofactor was included in the structural model.

For visualization, PyMOL 2.5 Molecular Graphics System was used. In order to visualize how membrane proteins are positioned in the membrane, PPM 3.0, which provides spatial arrangements of membrane proteins within the lipid bilayer, was employed [39]. For Poisson–Boltzmann electrostatic potential calculations, APBS (Adaptive Poisson–Boltzmann) was used to analyze the electrostatic properties of macromolecules. When integrated with PyMOL, APBS enables the visualization of these calculations as an electrostatic potential mapped onto the molecular surface, providing insights into the charge distribution and electrostatic environment of the molecule.

The model is available in ModelArchive at https://www.modelarchive.org/doi/10.5452/ma‐ydyj6

## Results

### Structure prediction of the recombinant cytochrome Cyc2_*TAG1_1*_

Based on AlphaFold3 predictions, Cyc2_TAG1_1_ is predicted to adopt a β‐barrel topology, formed by 16 antiparallel β‐strands arranged into a cylindrical architecture and fused to a heme *c* domain at the N‐terminal characterized by the conserved CXXCH motif in its sequence (Fig. 1A and Fig. S1). Porins usually have both the N and C termini in the periplasmic area, as well as extended extracellular loops and short periplasmic twists [40]. According to this conventional orientation, cytochrome Cyc2's heme domain should be located on the barrel's periplasmic side, which is consistent with its probable interaction with the periplasmic soluble electron carriers unidentified until now. However, its precise position within the barrel remains unknown. Figure 1B presents the calculated spatial arrangement of the protein relative to the lipid bilayer based on this orientation. An estimated hydrophobic thickness of 23.5 ± 1.1 Å indicates that the β‐barrel region of cytochrome Cyc2_TAG1_1_ is properly embedded within and in contact with the membrane lipids. The predicted small tilt angle of 2° ± 1° further suggests a correct, stable, and energetically favorable insertion of the β‐barrel into the membrane. An annular cavity is predicted within the protein with approximate dimensions of 20 Å (depth) × 20 Å (diameter), with an acidic surface at the inside toward the heme‐binding pocket (Fig. 1B,C). The negatively charged amino acids could either be involved in Fe(II) binding presenting an electron transfer mechanism as suggested by Jiang *et al*. [41] and/or this negatively charged surface simply binds and complexes Fe(III) and thus prevents Fe(III) from precipitation before it exits the barrel [42].

**Fig. 1:**
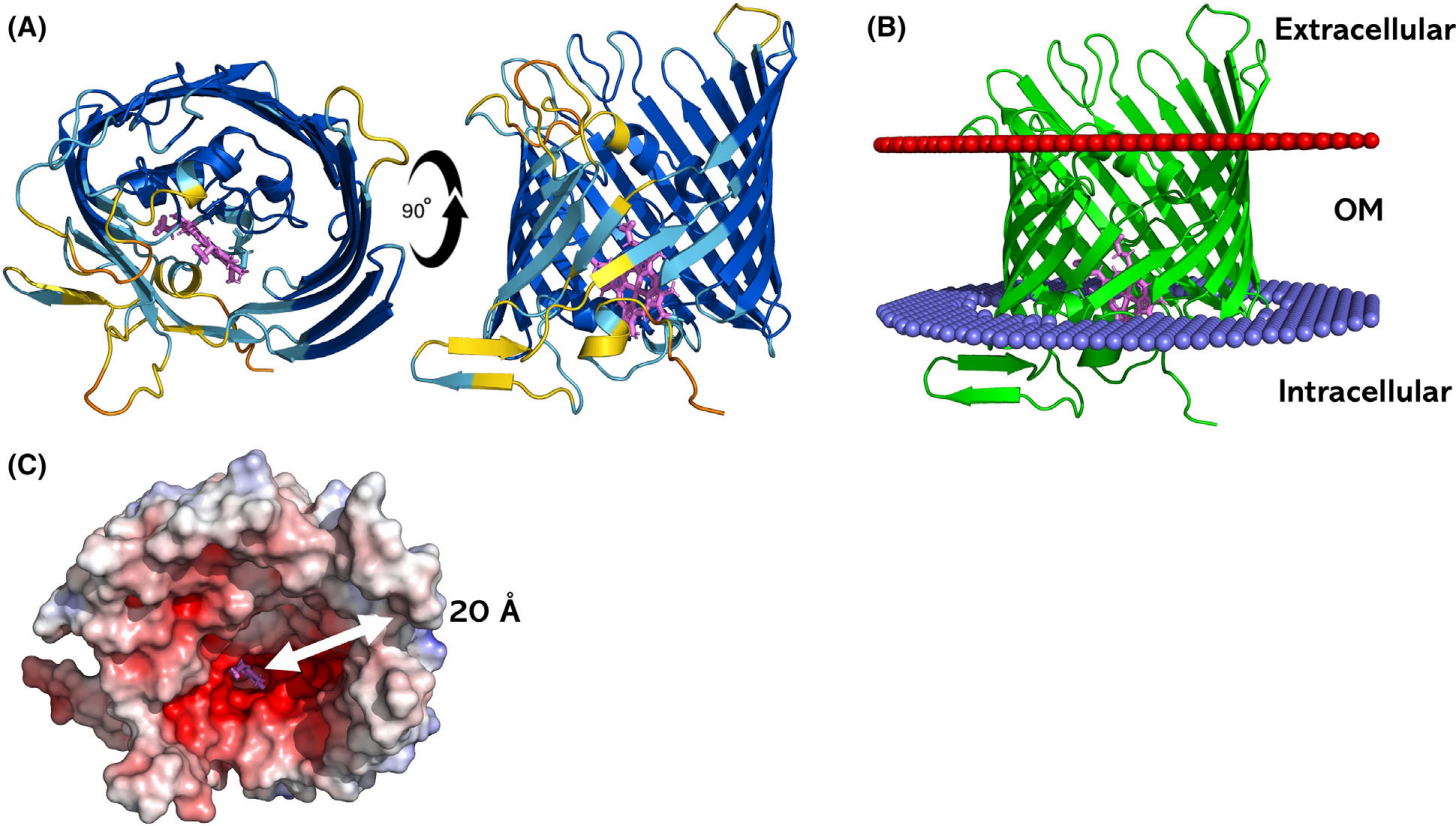
(A) Ribbon diagram of the AlphaFold model0 Cytochrome Cyc2_TAG1_1_. The figure shows the three‐dimensional structure of the Cytochrome Cyc2_TAG1_1_, displayed in two orientations (rotated by 90°). The protein backbone is colored according to the AlphaFold3 predicted Local Distance Difference Test (pLDDT) confidence score. The model was generated using the mature protein sequence starting at residue 23, after removal of the predicted N‐terminal signal peptide (residues 1–22) identified using the SignalP server. The heme c cofactor was included in the structural model. AlphaFold (AF3) predicted score colors: Yellow = poor confidence, orange = low confidence, and a blue gradient (light to dark) = increasing confidence, with darker blue indicating the highest confidence. Heme c is shown in pink. The model is available in ModelArchive at https://www.modelarchive.org/doi/10.5452/ma‐ydyj6. (B) Cyc2_TAG1_1_ arrangement in lipid bilayer viewed from the side, where the upper red disk represents phospholipid heads on the outer membrane facing the extracellular matrix and the bottom blue disk represents the phospholipids' polar heads facing the periplasm. (C) Computational model and electrostatic potential of the outer membrane cytochrome Cyc2_TAG1_1_ where blue indicates regions of positive potential, whereas red depicts negative potential.

### Cyc2_TAG1_1_ heterologous production and purification

*Ghiorsea bivora*'s cytochrome Cyc2_TAG1_1_ was heterologously produced as an outer membrane‐associated fusion protein in *E. coli* BL21(DE3) harboring the *ccm* genes required for heme maturation. Expression in the presence of ferric iron, to help heme iron maturation, in the culture medium yielded a holoprotein with an apparent molecular weight of 45 kDa, as confirmed by TMBZ staining on SDS/PAGE (Fig. 2A, lane 2).

**Fig. 2:**
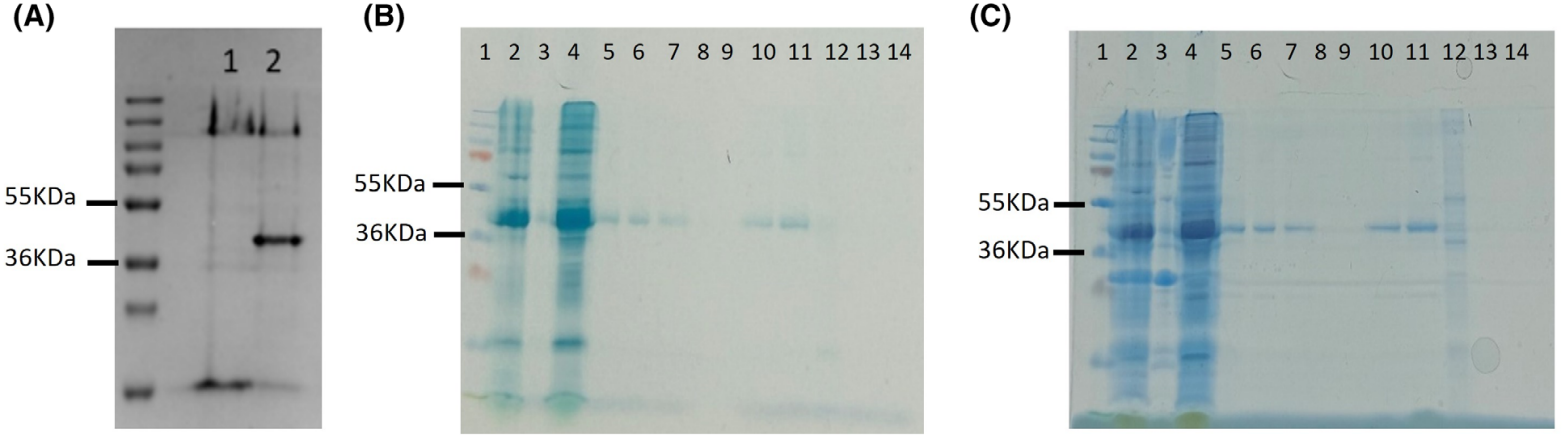
SDS polyacrylamide gel of cytochrome Cyc2_TAG1_1_ production, solubilization and purification (A): 3,3′,5,5′‐Tetramethylbenzidine (TMBZ) Heme‐stained sodium dodecyl sulfate–polyacrylamide gel electrophoresis (SDS/PAGE) showing cytochrome Cyc2_TAG1_1_ expression in *E. coli* BL21 before induction (lane 1) and after 18 h of induction with Isopropyl‐β‐D‐1‐thiogalactopyranoside (IPTG) (lane 2). (B): TMBZ heme‐stained gel of cytochrome Cyc2_TAG1_1_ solubilization and His tag purification. (C): Instant Blue stained gel. For (B) and (C) lane 1: ladder, lane 2: outer membrane enrichment, lane 3: pellet insoluble after solubilization, lane 4: soluble fraction with N‐Laurylsarcosine (NLS) = input, lane 5–6–7: eluted fractions containing cytochrome Cyc2_TAG1_1_, lane 10–11: cytochrome Cyc2_TAG1_1_ fractions after detergent exchange, lane 12: flow through, lane 13–14: concentrator flow through.

Differential centrifugation was used to recover the outer membrane fraction according to their density [43]. For cytochrome Cyc2_TAG1_1_ solubilization, several detergents and conditions have been tested (data not shown), leading to the choice of the anionic detergent N‐lauroyl sarcosine (NLS) as the most efficient detergent for solubilization. After 1 h of solubilization, most of the outer membrane fraction including the band corresponding to cytochrome Cyc2_TAG1_1_ appeared in the soluble fraction (Fig. 2BC lane 4). The histidine tagged recombinant cytochrome Cyc2_TAG1_1_ was purified via Ni‐NTA affinity chromatography. A major band, with an apparent molecular weight of approximately 45 kDa, was observed in the eluted fractions on SDS/PAGE (Fig. 2C lanes 5–6–7) and tested positive for TMBZ staining (Fig. 2B, lanes 5–6–7). This band has been identified by mass spectrometry as cytochrome Cyc2_TAG1_1_ (Table S1). Detergent exchange was then performed from NLS to the nonionic detergent N‐dodecyl‐β‐D‐maltoside (DDM), known to preserve activity and stability of transporters, receptors and channels [44] (Fig. 2B,C, lanes 10–11). Although the final purification step exhibits minor contaminant bands (Fig. 2C, lanes 10 and 11), the yield (around 68 μg per liter culture) and overall purity of the target protein (93% of the total protein) are sufficient for subsequent biochemical characterization.

Purified cytochrome Cyc2_TAG1_1_ exhibits a distinctive heme Soret peak of 408 nm and can be reduced with sodium dithionite (Fig. 3), demonstrated by the shift of the Soret peak from 408 nm to 418 nm and the appearance of beta and alpha peaks at 524 nm and 552 nm, respectively, corresponding to a low‐spin c‐type cytochrome [45, 46]. The addition of potassium ferricyanide as an oxidizing agent to the purified cytochrome Cyc2_TAG1_1_ did not induce any change in the spectrum, indicating that Cyc2_TAG1_1_ was in the oxidized state as prepared. These assays demonstrated that purified cytochrome Cyc2_TAG1_1_ had effectively inserted a heme group, is redox active, and could have two axial ligands, allowing us to test its Fe(II) oxidation capacity.

**Fig. 3:**
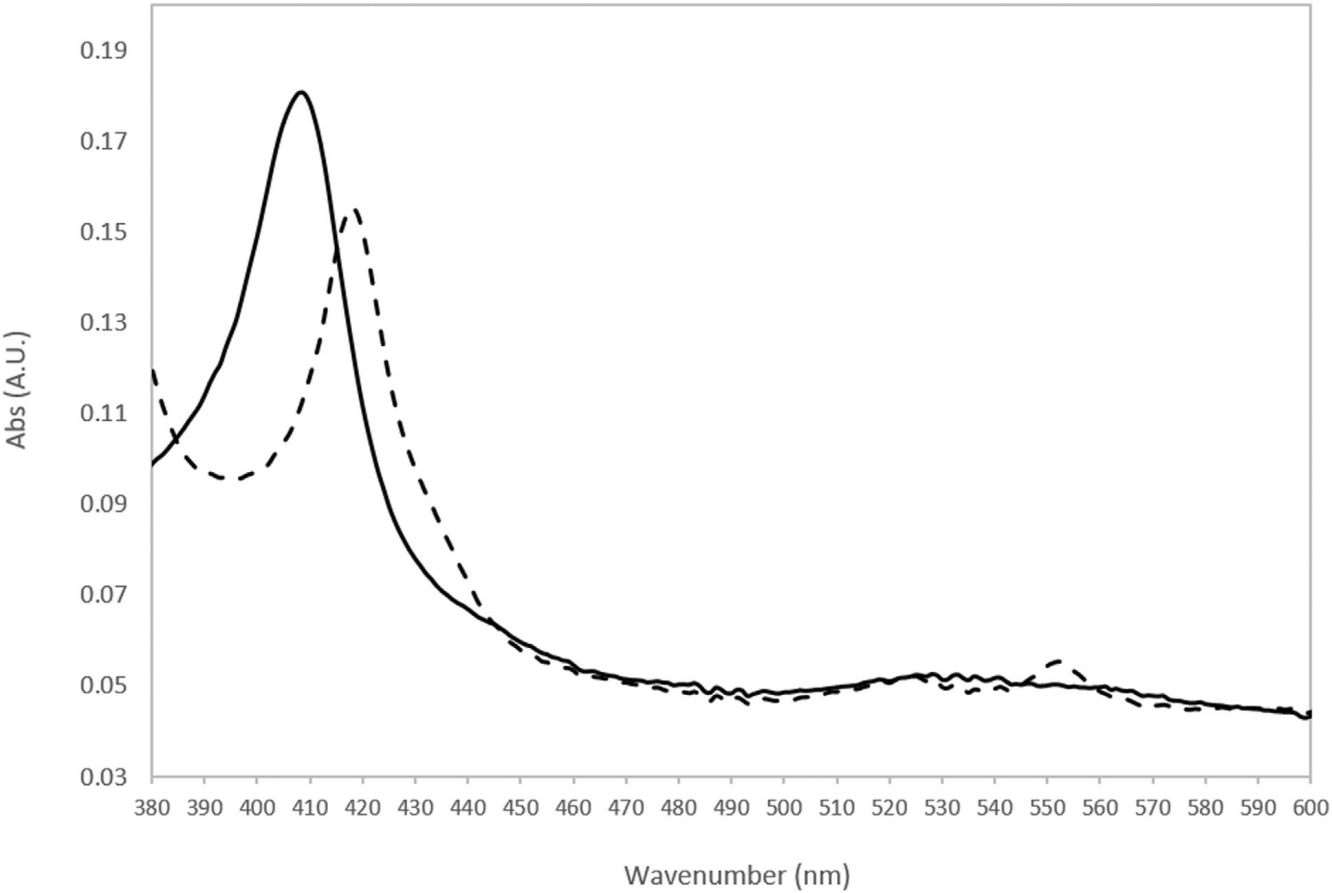
UV–Vis spectra of the purified Cyc2_TAG1_1_ in 50 mm HEPES (4‐(2‐Hydroxyethyl)‐1‐piperazineethanesulfonic Acid) pH 7 in the oxidized form (Solid black) or Sodium dithionite reduced state (dashed black).

### Validation and characterization of cytochrome Cyc2_TAG1_1_ iron oxidase activity *in vitro*

An iron oxidation assay was developed using DCPIP as an artificial terminal electron acceptor in the presence of Fe(II) sulfate (FeSO₄) as dissolved Fe(II) source and Cyc2_TAG1_1_. Performed under anaerobic conditions, this assay consists of 2 reactions: the oxidation of ferrous iron by the cytochrome Cyc2_TAG1_1_, and the reoxidation of the Cyc2_TAG1_1_ by DCPIP. Once reduced, the DCPIP becomes colorless leading to the decrease in the absorbance that was followed at 522 nm [47, 48]. In this study, the chemical fate of iron or the formation of solid ferric phases was not assessed as it was beyond the scope of the present work. To measure Fe(II) oxidation activity of cytochrome Cyc2_TAG1_1_ and given that DCPIP can oxidize Fe(II), we measured the initial velocity rates (v₀) of DCPIP chemical reduction with 100 μm of FeSO₄ in the absence of Cyc2_TAG1_1_. Cyc2_TAG1_1_ addition resulted in an increase in the initial reaction rate corresponding to a twofold increase of the initial rate. Cytochrome Cyc2_TAG1_1_'s iron‐oxidizing activity and its quantification were confirmed by velocity differences between both the chemical control and the enzymatic assay. Additionally, the direct reaction of DCPIP with Cyc2_TAG1_1_ alone was checked and no reduction of DCPIP was detected (Fig. 4 and Table S2).

**Fig. 4:**
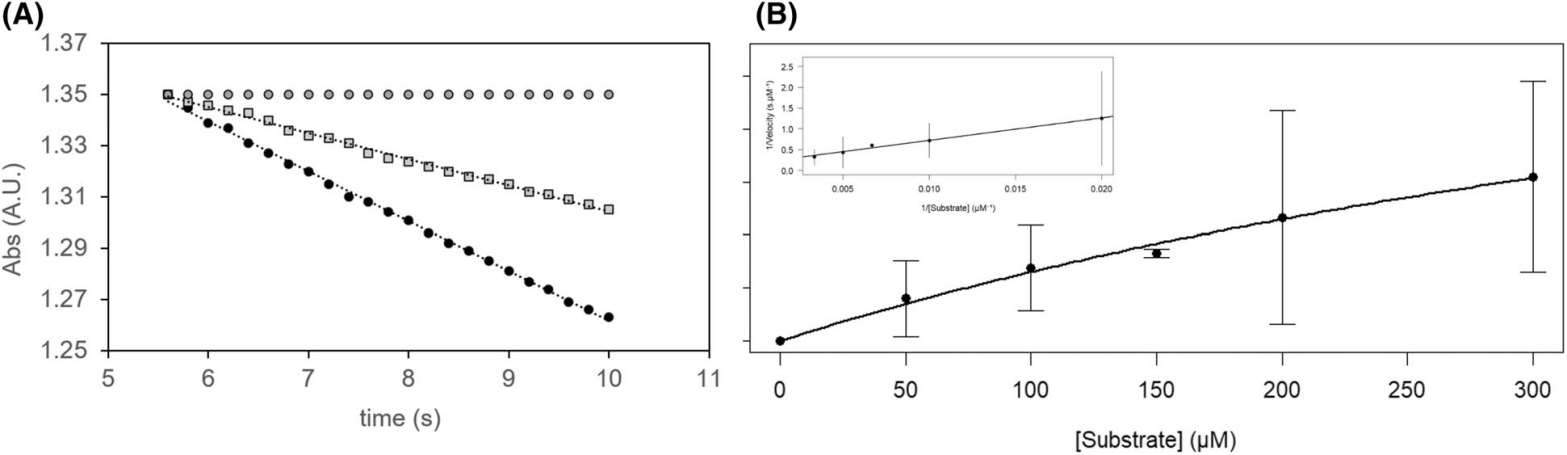
Kinetic measurement of 2,6‐Dichlorophenolindophenol (DCPIP) reduction by Cyc2_TAG1_1_. (A): Control reaction of DCPIP with Cyc2_TAG1_1_ (gray spheres) showing no DCPIP reduction. DCPIP chemical reduction by 100 μm Fe^2+^ in the absence of Cyc2_TAG1_1_ (gray squares) displays a decrease in absorbance over time, indicating a slow rate of Fe(II) oxidation by DCPIP. DCPIP reduction by 100 μm Fe(II) in the presence of Cyc2_TAG1_1_ (black spheres) used for initial velocity rate measurement resulted in a steeper decline in absorbance. (B) Michaelis–Menten nonlinear regression of cytochrome Cyc2_TAG1_1_ mediated Fe(II) oxidation using DCPIP as electron acceptor. Data points represent mean values ± SD of triplicate measurements. Inlet: Lineweaver–Burk plot of cytochrome Cyc2_TAG1_1_ activity using Fe(II) as the electron donor and DCPIP as the electron acceptor.

To quantitatively assess cytochrome Cyc2_TAG1_1_ activity under experimental conditions, we determined its apparent kinetic parameters (V_max_ and K_m_) for Fe(II) oxidation by steady state analysis using the Michaelis–Menten model. Given that the Fe(II) concentration range did not fully encompass substrate saturation and that the fitted parameters are associated with substantial uncertainty, the calculated Km and Vmax values are considered apparent estimates rather than absolute kinetic constants. Both nonlinear regression and Lineweaver–Burk linearization yielded consistent orders of magnitude estimates (Fig. 4). The parameter estimates reported here were obtained using nonlinear regression which are less prone to artifacts and provide statistically more reliable parameter estimates. In the presence of DCPIP as an electron acceptor, kinetic analysis showed an apparent K_m_ of 627 ± 226 μm and an apparent V_max_ of 19 ± 5 μM·s^−1^ for iron sulfate (Fig. 4). The enzyme exhibited an apparent k_cat_ of 633 s^−1^ ± 167 s^−1^ and a specific activity of 905 μmol·min^−1^·mg^−1^ ± 238 μmol·min^−1^·mg^−1^. Its catalytic efficiency (k_cat_/ K_m_ = 10 × 10^5^ m ^−1^·s^−1^) indicates efficient iron‐oxidizing activity [49, 50].

### Redox potential of Cyc2_TAG1_1_

Having shown that Cyc2_TAG1_1_ can oxidize iron sulfate in the presence of a final electron acceptor and in order to progress in the characterization of this enzyme, knowledge of the redox midpoint potential of the heme is important. To this end, we performed a redox titration of the protein at pH 7. Figure 5 shows the absorption changes at 418 nm as a function of the redox potential that can be fitted to an *n* = 1 Nernst curve with a redox midpoint potential of −130 mV *vs* SHE. We estimate the cumulated error inherent to our method to +/−20 mV [51, 52].

**Fig. 5:**
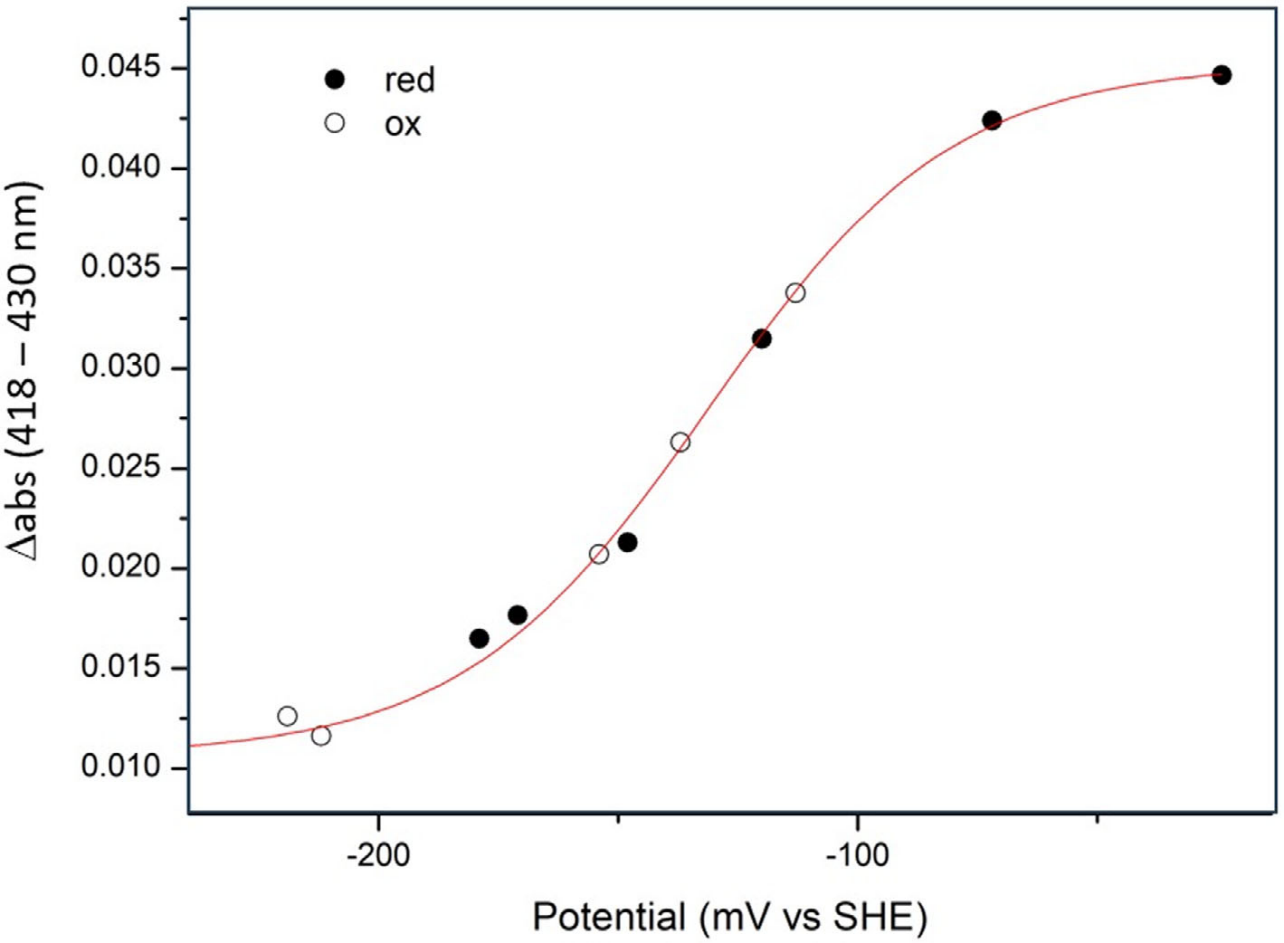
Redox titration. (A) Absorbance changes of Cyc2_TAG1_1_ at 418–430 nm as obtained during a redox titration at pH 7 in reductive (black dots) and oxidative (open dots) directions. The line represents a fit to a single Nernst equation with *n* = 1 and Em = −130 mV.

### EPR

To characterize the ligand environment of the heme cofactor, we recorded Electron Paramagnetic Resonance (EPR) spectra of the enriched cytochrome Cyc2_TAG1_1_ sample (nondialyzed) and the outer membrane enrichment. Purified Cyc2_TAG1_1_ displayed two characteristic signals of heme c center with g‐values at g_z_ = 2.94 and g_y_ = 2.25 corresponding to a low‐spin cytochrome *c* heme center with two axial ligands either histidine or methionine consistent with a nearly parallel orientation to the heme iron [53, 54, 55, 56]. A slight broadening of *g* = 2.95 to the lower g‐value was seen in the outer membrane enrichment with the presence of a very small *g* = 6 signal which may account for additional heme protein recovered with the outer membrane (Fig. 6). Besides the characteristic low‐spin heme *c* signals, weak resonances were detected at *g* = 2.0 and *g* = 4.3 in both samples. The *g* = 2.0 signal likely originates from a minor paramagnetic species (e.g., a free radical or presence of adventitious Fe(III)), while the *g* = 4.3 signal is characteristic of rhombically distorted high‐spin Fe(III) and is attributed to a minor ferric species or trace iron‐containing impurities in the sample [57, 58, 59]. The resulting spectra of both the purified Cyc2_TAG1_1_ and the outer membrane fraction are superposable, meaning that the observed EPR features are intrinsic to Cyc2_TAG1_1_ and are not significantly affected by detergent solubilization or the presence of imidazole during purification procedure.

**Fig. 6:**
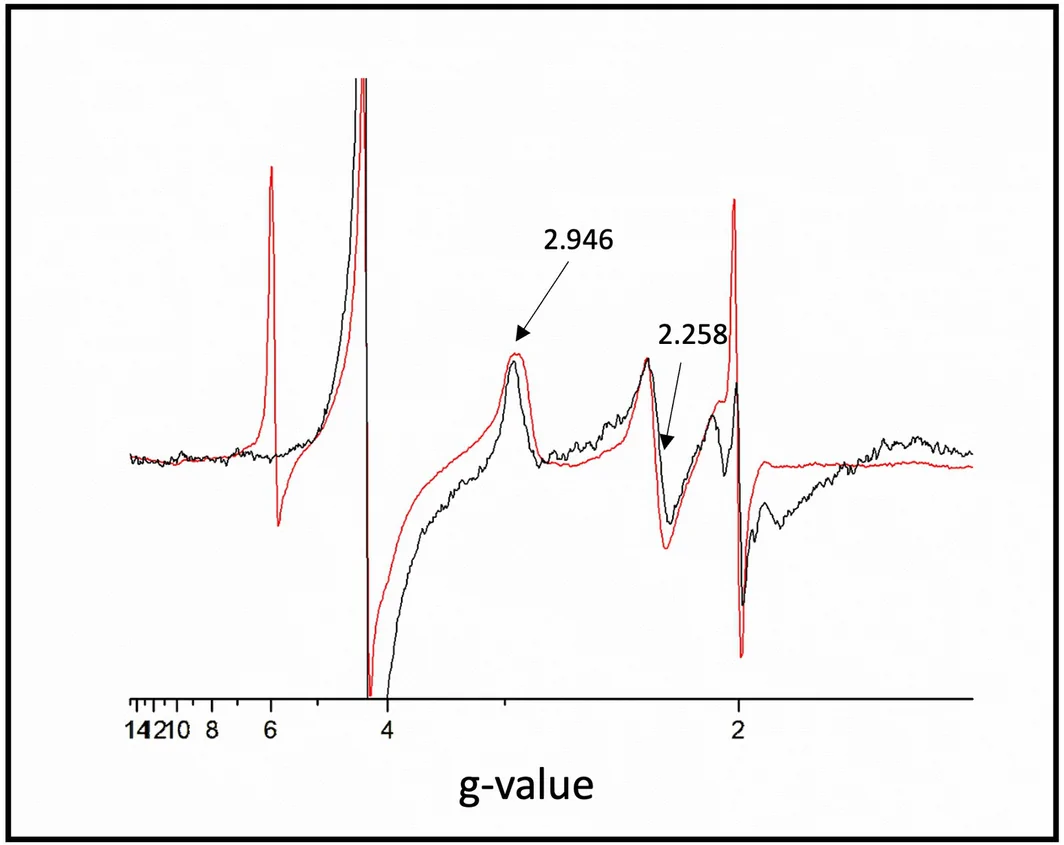
X‐band Electron Paramagnetic Resonance (EPR) spectra of purified Cyc2_TAG1_1_ (in black) and outer membrane enrichment (in red) recorded at 15 K with a microwave power of 6.5 mW, a microwave frequency of 9.468 GHz and a modulation amplitude of 3.15 mT. The two signals' g‐value (2.946 and 2.258) correspond to the signal of the two axial ligands.

## Discussion

In the present study, we report the heterologous production of the *G. bivora* cytochrome Cyc2_TAG1_1_ in the outer membrane of *E. coli*, followed by its purification, iron‐oxidizing activity assessment in the presence of an artificial electron acceptor, a biophysical characterization by EPR spectra and redox properties. Our study provides the first quantitative kinetic characterization of a Cyc2 homolog. Using the same gene construct as previously described by Keffer *et al*. [25], we have implemented critical refinements in both solubilization and purification protocols. These methodological modifications have enabled the recovery of Cyc2_TAG1_1_ with minimal contamination by other proteins, thereby eliminating the requirement for high sucrose concentrations for solubilization that impede efficient dialysis and compromise sample homogenization during activity assays. This enhanced purity of our enzyme preparation significantly improves the reliability of activity measurements and simplifies biophysical characterization. Our activity assay was designed to include an artificial electron acceptor, DCPIP, and FeSO₄ as the source of dissolved Fe(II), enabling the investigation of Cyc2_TAG1_1_‐mediated Fe(II) oxidation under controlled laboratory conditions. In the present work, FeSO₄ was selected as a soluble and well‐characterized Fe(II) source to facilitate direct assessment of Cyc2_TAG1_1_ enzymatic activity since it was already used in previous studies investigating Cyc2‐mediated iron oxidation; notably, studies on the Cyc2 from the acidophilic *A. ferrooxidans* [19] and kinetic characterization of its iron:rusticyanin oxidoreductase [60]. Cyc2_TAG1_1_ exhibited an apparent K_m_ of 627 ± 226 μm for iron sulfate which falls within the same range as the soluble acidic‐stable iron:rusticyanin oxidoreductase from *A. ferrooxidans*, reported to be approximately 300 μm for iron sulfate [60]. Velocity results led to an apparent V_max_ of 19 ± 5 μM·s^−1^ indicating that cytochrome Cyc2_*TAG1_1*_ accelerates electron transfer from Fe(II) to DCPIP, supporting its role as a catalyzer for Fe(II) oxidation. The observed variability in our kinetic parameters estimates is likely influenced by the use of detergents and the necessity to prevent abiotic iron oxidation by working in an anaerobic chamber and preparing our different buffers anaerobically. Despite working with minimal detergent concentration, essential to stabilize Cyc2_TAG1_1_ for upstream experiments (two‐ to threefold the critical micellar concentration (CMC)) [61, 62], it can introduce variability by altering substrate accessibility and diffusion through substrate partitioning and micelle formation. All of that may impact apparent catalytic efficiency and apparent substrate affinity. To lessen these issues, all experiments were performed within an anaerobic chamber, which while ensuring data relevance, introduces additional technical variability such as manual mixing.

Given the lack of prior biochemical characterization in the literature, the kinetic parameters obtained for this enzyme underscore the novelty of our findings and provide the first experimental basis for generating hypotheses regarding its potential role in iron metabolism. The moderate apparent K_m_ value (627 ± 226 μM), despite its variability, may suggest that the enzyme requires relatively high iron sulfate concentrations to achieve half‐maximal activity under the experimental conditions used. Notably, the apparent K_m_ of cytochrome Cyc2_TAG1_1_ falls within the range of dissolved iron concentrations reported for hydrothermally influenced environments inhabited by Zetaproteobacteria, raising the possibility that its kinetic properties are compatible with these iron‐rich settings. For example, Fe(II) concentrations of 3800–5000 μm have been reported at TAG vents [63], 70–2800 μm at the Lucky Strike vent field [64, 65], and up to 20 mm range at the Fåvne vent field along the Arctic Mid‐Ocean Ridge [14], all sites where *G. bivora* has been detected [10, 12, 14]. These environments are therefore generally consistent with conditions that can support Cyc2_TAG1_1_‐mediated Fe(II) oxidation. In contrast, dissolved Fe(II) concentrations in open‐ocean, nonhydrothermally influenced waters can be orders of magnitude lower (down to ~ 0.1 nm in the deep Atlantic Ocean) [63]. Under such conditions, substrate availability would be expected to limit Cyc2_TAG1_1_ activity. Overall, these comparisons are consistent with the recognized ecological niche of Zetaproteobacteria, which colonize Fe(II)‐enriched environments, such as hydrothermally influenced or anthropogenically impacted habitats, rather than Fe(II)‐depleted seawater. The enzyme also demonstrates an effective catalytic performance under experimental conditions, as indicated by its high apparent V_max_ (19 ± 5 μM·s^−1^) and turnover number K_cat_ (633 ± 167 s^−1^). Together with its high apparent specific activity (905 ± 238 μmol·min^−1^·mg^−1^), these results indicate that Cyc2_TAG1_1_ is capable of rapid Fe(II) oxidation under the experimental conditions. Importantly, the catalytic efficiency (1.0 × 10^6^ m ^−1^·s^−1^) places Cyc2_TAG1_1_ among efficient iron oxidases [50].

Cytochrome Cyc2_TAG1_1_ redox potential was determined to be −130 ± 20 mV versus SHE, which is the lowest redox potential measured for cytochrome Cyc2 homologs. For comparison, Cyc2_PV‐1_ redox potential at pH 8 is 208 ± 20 mV [25] while the cytochrome Cyc2 from *A. ferrooxidans* ranges from 560 mV at pH 4.8 to around 700 mV at pH 2 [19, 66], this high redox potential is largely attributed to its extremely acidic pH environment, which shifts the Fe(III)/Fe(II) redox couple to more positive values. Nevertheless, the redox potential of Cyc2_TAG1_1_ falls within the range reported for MtoA, the decaheme c‐type cytochrome involved in Fe(II) oxidation in freshwater neutrophilic iron‐oxidizing bacteria, *Sideroxydans lithotrophicus*, whose 10 hemes span a broad range of redox potentials from approximately +100 to −400 mV versus SHE [67]. This suggests that low‐potential hemes can be compatible with Fe(II) oxidation and that the relationship between redox potential and iron‐oxidizing activity may be more complex than predicted from thermodynamic considerations alone. Further comparative biophysical and structural studies are needed to understand this observed difference in redox potential. Interestingly, it was recently demonstrated that the exometabolome of *G. bivora* TAG1 grown on iron, but not on hydrogen, markedly inhibits the abiotic oxidation of ferrous iron, resulting in sustained Fe(II) accumulation. Under oxic conditions, *G. bivora* produces extracellular metabolites that increase the Fe(II) half‐life approximately 100‐fold, from ~ 3 to ~ 335 days. These ligands may modify the redox chemistry and availability of extracellular iron species, suggesting that *G. bivora* actively regulates its extracellular micro‐environment to preserve Fe(II) availability [68].

Taken together, these results reveal redox diversity among Cyc2 homologs and other iron‐oxidizing proteins, suggesting that evolutionary tuning of cytochrome potentials may allow iron oxidizers to exploit a broad range of geochemical niches.

To conclude, this study provides a first step toward the biochemical characterization of Cyc2_TAG1_1_. Recombinant production and purification of Cyc2_TAG1_1_ from the outer membrane fraction of *E. coli* allowed the assessment of its Fe(II)‐oxidizing activity, the determination of its apparent catalytic parameters, the elucidation of its spectroscopic and redox properties through EPR spectroscopy and redox potential measurements. These results provide new insights into Cyc2_TAG1_1_ catalytic properties, supporting its proposed role in Fe(II) oxidation in iron‐oxidizing bacteria. They also provide a foundation for future studies aimed at identifying its native redox partner(s) and characterizing its activity under more physiologically relevant conditions.

## Conflict of interest

The authors declare no conflicts of interest.

## Author contributions

JA conducted the investigation, developed the methodology, performed the formal analysis, and wrote the original draft; AD supervised the biochemical aspects, validated the findings, and contributed to the writing by reviewing and editing; FB provided resources for redox potential measurement, validated the results, and contributed to the writing by reviewing and editing; CR supervised the study on Zetaproteobacteria and iron‐rich mat ecology, validated the work, acquired funding, and contributed to the writing by reviewing and editing; LE contributed resources for membrane protein purification, developed the methodology; HG conceptualized the study, supervised the project, acquired funding, and contributed to the writing by reviewing and editing; MN conceptualized the study, supervised the project, and contributed to the writing by reviewing and editing.

## Supporting information


**Table S1,** Unique peptides matching Cyc2_TAG1_1_.
**Table S2**. Initial reaction rate of DCPIP reduction under different conditions.
**Table S3**. M9 2x media composition.
**Fig. S1**. Fasta sequence of Cyc2_TAG1_1_ NCBI Reference Sequence.
**Fig. S2**. Redox titration of Cyc2_TAG1_1_ monitored by UV–visible spectroscopy.
**Data S1**. code R for kinetics parameters.

## Data Availability

All data supporting the findings of this study are available within the article and its Supporting Information. The protein structure model is publicly available in ModelArchive at https://www.modelarchive.org/doi/10.5452/ma‐ydyj6. The custom script used to fit the experimental data is provided as part of the Supporting Information.
