# Supplementary material for: Characterization of the key membrane protein cytochrome Cyc2 from Ghiorsea bivora
Source: FEBS Open Bio. 2026 Oct 23:10.1002/2211-5463.70350. Online ahead of print. doi: 10.1002/2211-5463.70350 (PMC13692758; doi:10.1002/2211-5463.70350)
Supplement: Supplementary file 1 [file FEB4-9999-0-s001.pdf]

Supplementary Information:

Characterization of the key membrane protein Cytochrome Cyc2 from *Ghiorsea bivora*

Azizi Josiane[1,3], Dolla Alain[1], Baymann Frauke[2], Rommevaux Céline[1], Elantak Latifa[3], Gaussier Hélène[1] , Nouailler Matthieu[3]

Supplementary Table 1 : Unique peptides detected by tandem MS/MS that matched to $Cyc2_{TAG1\_1}$ with high confidence. Individual peptides identified cover 34% of the entire amino acid sequence of $Cyc2_{TAG1\_1}$.There are zero missed cleavages.

Sequence	# PSMs	Xcorr	Modifications
QTGSACLACHFQSFPTLNAYGR	5	6.82	2xCarbamidomethyl [C6; C9]
ATVKPTHNLIFLAGLGQDK	4	5.72	
TGIDVQAFGEVGDMQIGVFADYATAPK	4	4.52	1xOxidation [M14]
ANSIGFADQVFMVGGR	18	4.4	1xOxidation [M12]
SATNVYATATSDR	23	4.34	
ANSIGFADQVFMVGGR	2	4.08	
PTTSEAIPAFAR	2	3.16	
TSAFGVTPASK	19	2.73	
TGYSFR	4	1.93	
ADFTIDK	1	1.91	

Supplementary Table 2 : Initial reaction rate of DCPIP reduction under different conditions. The two first columns correspond to raw data of Cyc2$_{TAG1_1}$ average initial velocity and control for different Fe(II) concentrations. The last column corresponds to the corrected data after control substraction. The second line (no Fe(II)) correspond to the primary control showing no DCPIP reduction without electron donor.

[Fe(II)] (μM)	Raw v_0 (Cyc2TAG1), μM/s	Raw v_0 (control), μM/s	Corrected v_0 (Cyc2TAG1), μM/s
0	0	0	0
50	1.75 ± 0.71	0.95 ± 0.46	0.799 ± 0.71
100	3.16 ± 0.80	1.77 ± 0.29	1.38 ± 0.8
150	4.57 ± 0.07	2.92 ± 0.62	1.65 ± 0.07
200	4.86 ± 2	2.53 ± 0.53	2.32 ± 2
300	7.85 ± 1.8	4.75 ± 0.24	3.09 ± 1.8

Supplementary Table 3 : M9 2x media composition.

Composition for 1L	
Disodium Phosphate Heptahydrate	25.6 g/L
Monopotassium Phosphate	6 g/L
Sodium Chloride	1 g/L
Final pH (2X solution): 6.6–7.0 at 25°C	
40% D-Glucose solution	20 mL
1.0 M $MgSO_4$ solution	2 mL
1.0 M $CaCl_2$ solution	0.1 mL
Chloramphenicol 30 mg/mL	1 mL
Kanamycine 100 mg/mL	1 mL

(A)

> WP_038247608.1 hypothetical protein [Ghiorsea bivora]

MKKIILSAASLAVAAVASVSVAPTTSEAIPAFARQTGSACLACHFQSFPTLNAYGRAF
KMGALTDVGDQALIEDEGLSIPAVVNWTAMVRPQFQSVDNGTTKANSIGFADQVFMVG
GRGGEHTGVFIEFAAADVAGGGQPYANVQLFNSWDMGDMKVGATIWADGFGEDSGLQL
MSVWGQHGGLLGGKSLTINGAMAGLAGGVGVTGWVGTDAWAAQLAMVDVASGATGVGT
GFTLAPVLRGNYFLEAGDWEVGLGAIAVTGSQNNIDTKRTGIDVQAFGEVGDMQIGVF
ADYATAPKSATNVYATATSDRTGYSFRATVKPTHNLIFLAGLGQDKADFTIDKTLVGV
EWEAYQNFVVSLSYNEEKTSAFGVTPASKTKTTLLDFELLI

(B)

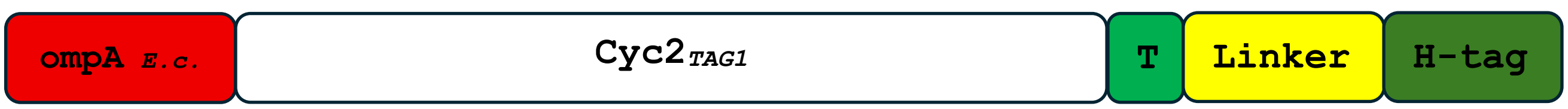

MKKTAIAIAVALAGFATVAQAPTTSEAIPAFARQTGSACLACHFQSFPTLNAYGRAFK
MGALTDVGDQALIEDEGLSIPAVVNWTAMVRPQFQSVDNGTTKANSIGFADQVFMVGG
RGGEHTGVFIEFAAADVAGGGQPYANVQLFNSWDMGDMKVGATIWADGFGEDSGLQLM
SVWGQHGGLLGGKSLTINGAMAGLAGGVGVTGWVGTDAWAAQLAMVDVASGATGVGTG
FTLAPVLRGNYFLEAGDWEVGLGAIAVTGSQNNIDTKRTGIDVQAFGEVGDMQIGVFA
DYATAPKSATNVYATATSDRTGYSFRATVKPTHNLIFLAGLGQDKADFTIDKTLVGVE
WEAYQNFVVSLSYNEEKTSAFGVTPASKTKTTLLDFELLI**ENLYFQSGGSGGSSHHHH**
HHH

Supplementary Figure 1 : (A) Fasta sequence of Cyc2$_{TAG1_1}$ NCBI Reference Sequence: WP_038247608.1, in bold : Predicted hydrophobic region of the signal Peptide (Sec/SPI) by the software SignalP-6.0 (B) Expressed construct used in this study, red : *E. coli* ompA signal sequence, black and underline : Cyc2$_{TAG1_1}$ sequence residus 23-389, used for the alphafold prediction , green : cleavage site of Tobacco etch virus (TEV) protease, yellow : flexible linker, brown : Histidine tag.

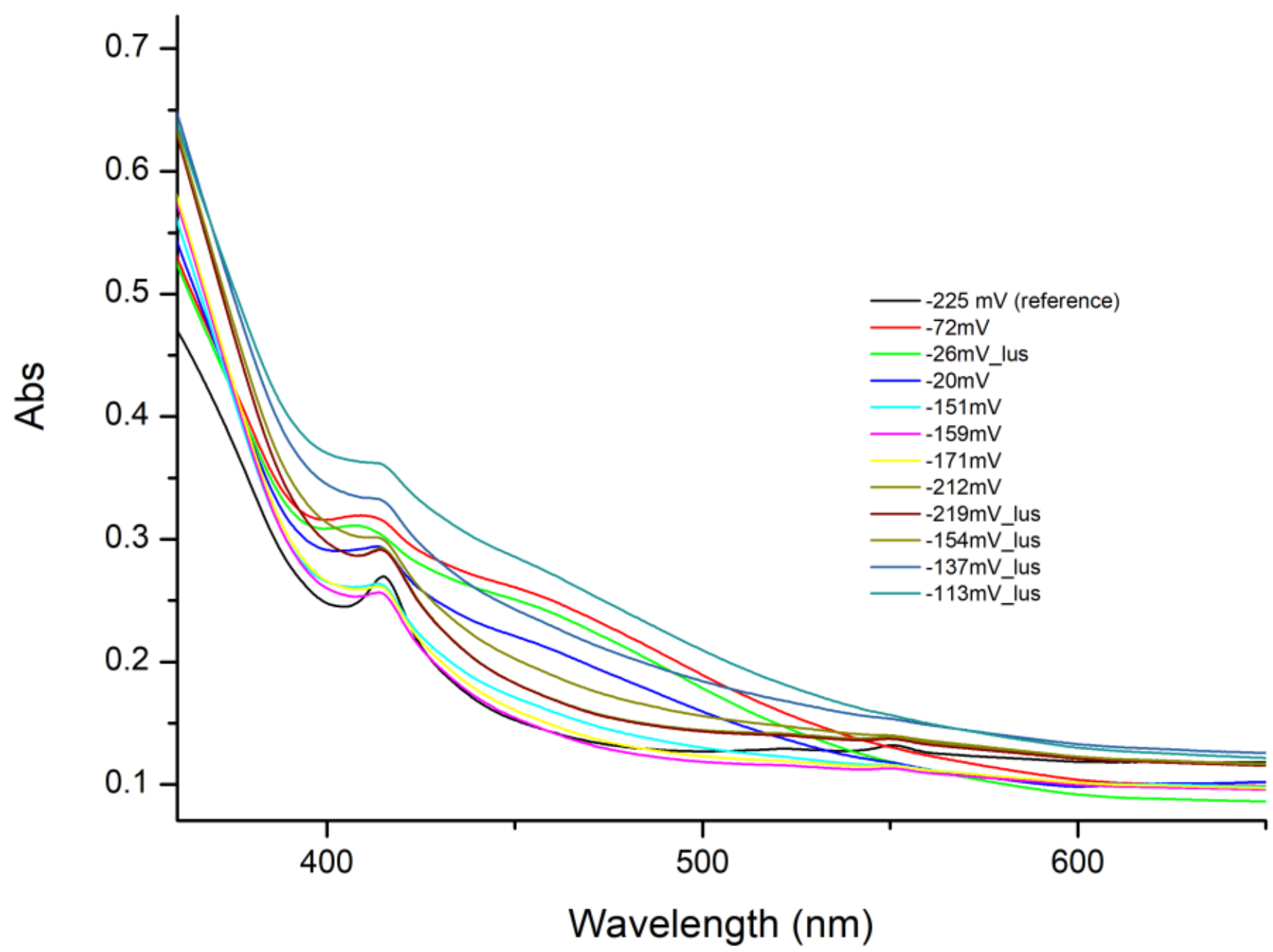

Supplementary Figure 2 : Redox titration of Cyc2$_{TAG1_1}$ monitored by UV–visible spectroscopy. UV–visible absorption spectra of Cyc2$_{TAG1_1}$ recorded at different redox potentials during anaerobic redox titration. The spectra show the progressive changes in the heme absorption features as the potential was increased from -225 mV (reference reduced state) to −20 mV. The spectral changes reflect the redox transition of the heme center from the ferric (Fe^{3+}) to the ferrous (Fe^{2+}) state.

Supplementary data 1 : code R for kinetics parameters.

```
# Nonlinear regression (Michaelis-Menten):
# Code used for nonlinear regression including the standard errors of each data point.

# 1. Data input (replace with your values)

substrate <- c(0, 50, 100, 150, 200, 300) # in µM

velocity <- c(0, 0.799019608, 1.387254902, 1.651960784, 2.328431373, 3.098039216) # in µM/s

se_velocity <- c(0, 0.719887615, 0.806011153, 0.074017985, 2.008703668, 1.803075516) # standard errors

# 2. Definition of the Michaelis-Menten model

model_MM <- function(substrate, Vm, Km) {
  Vm * substrate / (Km + substrate)
}

# 3. Nonlinear fitting of the model

fit_MM <- nls(velocity ~ model_MM(substrate, Vm, Km),
              start = list(Vm = max(velocity), Km = median(substrate)))

# 4. Extraction of parameters and standard errors

Vm <- coef(fit_MM)["Vm"]
Km <- coef(fit_MM)["Km"]

se_Vm <- summary(fit_MM)$parameters["Vm", "Std. Error"]
se_Km <- summary(fit_MM)$parameters["Km", "Std. Error"]
```

```
cat("\nEstimation by nonlinear regression (Michaelis-Menten):\n")
cat("Vm:", round(Vm, 3), "±", round(se_Vm, 3), "µM/s\n")
cat("Km:", round(Km, 3), "±", round(se_Km, 3), "µM\n")

# 5. Visualization with error bars

plot(substrate, velocity,
     xlab = "[Substrate] (µM)",
     ylab = "Velocity (µM/s)",
     main = "Nonlinear fitting (Michaelis-Menten)",
     pch = 19, las = 1,
     cex.lab = 1.1, cex.axis = 1.1,
     ylim = c(0, max(velocity + se_velocity) * 1.1))

# Add error bars
arrows(substrate, velocity - se_velocity,
       substrate, velocity + se_velocity,
       angle = 90, code = 3, length = 0.1, col = "black")

# Add fitted curve
curve(model_MM(x, Vm, Km),
      add = TRUE, col = "black", lwd = 2,
      from = min(substrate), to = max(substrate))

# Add legend
legend("topleft",
       legend = c("Experimental data", "Fitted model"),
       col = c("black", "black"),
       lty = c(NA, 1),
       pch = c(19, NA),

```

```
# Lineweaver-Burk regression results:
# Code used for Lineweaver-Burk regression including the standard errors of each data point.

# 1. Data input

substrate <- c(50, 100, 150, 200, 300) # in μM

velocity <- c(0.799019608, 1.387254902, 1.651960784, 2.328431373, 3.098039216) # in μM/s

se_velocity <- c(0.719887615, 0.806011153, 0.074017985, 2.008703668, 1.803075516) # standard errors


# 2. Calculation of inverses for Lineweaver-Burk

inv_substrate <- 1 / substrate # in μM⁻¹

inv_velocity <- 1 / velocity # in s/μM


# 3. Calculation of standard errors for inverses

se_inv_velocity <- se_velocity / (velocity^2)


# 4. Linear regression on inverses

fit_LB <- lm(inv_velocity ~ inv_substrate)


# 5. Extraction of coefficients and variance-covariance matrix

intercept_LB <- coef(fit_LB)["(Intercept)"]
```

```
slope_LB <- coef(fit_LB)["inv_substrate"]

vcov_LB <- vcov(fit_LB)


# 6. Calculation of Vm and Km

Vm_LB <- 1 / intercept_LB
Km_LB <- Vm_LB * slope_LB


# 7. Standard errors

se_intercept <- sqrt(vcov_LB["(Intercept)", "(Intercept)"])
se_slope <- sqrt(vcov_LB["inv_substrate", "inv_substrate"])
cov_intercept_slope <- vcov_LB["(Intercept)", "inv_substrate"]

se_Vm_LB <- se_intercept / (intercept_LB^2)

se_Km_LB <- sqrt(
  (Vm_LB^2 * se_slope^2) +
    (slope_LB^2 * se_Vm_LB^2) +
    (2 * slope_LB * Vm_LB * cov_intercept_slope)
)


# 8. Display results

cat("\n--- Lineweaver-Burk results ---\n")
cat("Vm:", round(Vm_LB, 3), "±", round(se_Vm_LB, 3), "µM/s\n")
cat("Km:", round(Km_LB, 3), "±", round(se_Km_LB, 3), "µM\n")
```

```
# 9. Visualization with error bars
plot(inv_substrate, inv_velocity,
     xlab = "1/[Substrate] (µM⁻¹)",
     ylab = "1/Velocity (s/µM)",
     main = "Lineweaver-Burk plot",
     pch = 19, las = 1,
     cex.lab = 1.1, cex.axis = 1.1,
     ylim = c(0, max(inv_velocity + se_inv_velocity) * 1.1))

# Error bars
for (i in 1:length(inv_substrate)) {
  segments(inv_substrate[i],
           inv_velocity[i] - se_inv_velocity[i],
           inv_substrate[i],
           inv_velocity[i] + se_inv_velocity[i],
           col = "black", lwd = 1)
}

# Regression line
abline(fit_LB, col = "black", lwd = 2)

# Legend
legend("topleft",
       legend = c("Experimental data", "Linear regression"),
       col = c("black", "black"),
       lty = c(NA, 1),
```

```
      pch = c(19, NA),
```

Non Linear regression results :

```
> cat("Vm:", round(Vm, 3), "±", round(se_Vm, 3), "µM/s\n")
Vm: 9.496 ± 2.546 µM/s
> cat("Km:", round(Km, 3), "±", round(se_Km, 3), "µM\n")
Km: 627.369 ± 225.987 µM
```

Linear regression results :

```
> cat("Vm:", round(Vm_LB, 3), "±", round(se_Vm_LB, 3), "µM/s\n")
Vm: 5.633 ± 1.167 µM/s
> cat("Km:", round(Km_LB, 3), "±", round(se_Km_LB, 3), "µM\n")
Km: 305.734 ± 65.705 µM
```
